# A Mixed Comparisons of Aerobic Training With Different Volumes and Intensities of Physical Exercise in Patients With Hypertension: A Systematic Review and Network Meta-Analysis

**DOI:** 10.3389/fcvm.2021.770975

**Published:** 2022-01-21

**Authors:** Zhenghui Lu, Yang Song, Hairong Chen, Shudong Li, Ee-Chon Teo, Yaodong Gu

**Affiliations:** ^1^Faculty of Sports Science, Ningbo University, Ningbo, China; ^2^Doctoral School on Safety and Security Sciences, Óbuda University, Budapest, Hungary; ^3^Faculty of Engineering, University of Szeged, Szeged, Hungary

**Keywords:** hypertension, exercise, blood pressure, high intensity intermittent, moderate-intensity aerobic exercise, network meta-analysis

## Abstract

It is essential for patients with hypertension to effectively reduce and maintain appropriate blood pressure levels. As one of the non-pharmacological and invasive methods, physical exercise seems to improve blood pressure of the patients with hypertension. However, different volumes and intensities of physical exercise on the improvement of hypertension are different. To understand the effects of the type of exercise training on blood pressure and the other health status of patients with hypertension, a network meta-analysis was used to compare the mixed effects of different types of exercise training. This systematic review includes all eligible randomized controlled trials of PubMed, Medline, Cochrane Library, and CINAHL. Twelve studies met the inclusion criteria (*n* = 846 participants at the end of the study). The results show that a medium-intensity training (MIT) is best in improving the blood pressure of patients with hypertension, while a high-volume high-intensity interval training (HVHIIT) is better in reducing body mass and resting heart rate. In addition, the analysis of the exercise capacity shows that HVHIIT has a better effect on the improvement of patients with hypertension. Noticeably, long-term high-volume and appropriate intensity exercise can effectively improve the health status of patients with hypertension. In short, for patients with high blood pressure, MIT seems to be better at lowering blood pressure, while HVHIIT can better improve exercise ability and physical fitness. However, larger randomized controlled trials with a longer duration than those included in this meta-analysis are needed to confirm these results.

## Introduction

The prevalence of hypertension is very high worldwide, and is the most common disease in primary care. It is commonly treated with chronic prescription drugs (1–4). Hypertension is a major risk factor for cardiovascular disease (5–8), and there is evidence that hypertension is a major cause of chronic kidney disease, dementia, and stroke (9–12). Obesity, unhealthy diet, lack of exercise, and alcoholism are all possible factors leading to high blood pressure (7, 13). There is ample evidence that there is a nearly linear relationship between body mass index (BMI) and blood pressure (14–17). It is reported that obese people (BMI > 30 kg/*m*^2^) account for more than 60% of the incidence of hypertension, and obese people are 3.5 times more likely to develop a hypertension (18, 19). Previous studies have shown that the prevalence of hypertension among adults has reached 40% (4, 20), and more than 7 million people die of high blood pressure each year (4, 14–16). A meta-analysis shows that when the systolic blood pressure (SBP) is > 115 mmHg or the diastolic blood pressure (DBP) is > 75 mmHg, the likelihood of cardiovascular events increases with the increase of blood pressure (21). For every increase in SBP (20 mmHg) or DBP (10 mmHg), the risk of fatal cardiovascular events doubled (22). Therefore, it is necessary to optimize the prevention and treatment of hypertension to reduce the morbidity and the mortality caused by related diseases.

Hypertension is either genetic or environmental factors related, or even both. Although the genetic susceptibility to hypertension cannot be changed, improving the lifestyle can significantly reduce the disease risk or improve hypertension (7). There is some controversy on the therapeutic effect of physical exercise (PE) as means in benefitting the health and improve chronic diseases (13, 23). As a non-drug treatment (24, 25), it has been proven by many studies that PE has a positive impact on the improvement of hypertension (4, 13, 23, 26–31). There is much evidence that both aerobic training and resistance training can improve systolic and diastolic blood pressure, and aerobic training seems to be superior to resistance training in reducing blood pressure (23, 32–34). However, some studies have shown that there may be differences between these two pieces of training (23). A review shows that aerobic and resistance training may have the same effect on blood pressure, but the potential physiological mechanisms are different (13). The effects of different intensities of exercise on people with hypertension may be different. Epidemiology shows that higher intensity of exercise can lower blood pressure (35), while too much high intensity of exercise may have adverse effects on the body (36). The different effects of exercise due to different capacities, types, frequencies, and times need further study.

In a recent meta-analysis (3), the effects of high-intensity interval training (HIIT) and medium-intensity training (MIT) on patients with hypertension were compared. The results showed that HIIT and MIT decreased SBP in patients with hypertension, and there was no significant difference between the two interventions in reducing SBP. The decrease of DBP was more significant in the HIIT group. In addition, the study also found that compared with the MIT group, the HIIT group also promoted the improvement of maximum oxygen uptake. It is worth noting that there were significant differences in the exercise capacity of the HIIT group included in the study in this meta-analysis. A review by Whitaker et al included 142 subjects in 7 studies (37). The results showed that compared with MIT, HIIT decreased the arterial blood flow velocity and response to CO_2_, and it also decreased the dynamic self-regulation phase, while significantly increased the deoxyhemoglobin compared with rest. However, the subjects included in this review are all healthy people, and the evidence for hypertensive people is still not very rich. Some studies have found that the high-volume HIIT seems to be more beneficial to weight loss, but there seems to be no difference in lowering blood pressure among 46 HIIT with different volumes (38, 39). However, although several studies have analyzed the effects of different volumes of HIIT on patients with hypertension, most studies have not clearly shown which volume and which type of exercise can minimize the blood pressure. Accordingly, we assume that different volumes and intensities of physical exercise may affect the blood pressure and health status of people with hypertension, and there is an optimal intervention plan. This review will study the effects of different volumes of HIIT, MIT, general physical activity, and blank on people with hypertension.

Human blood pressure is affected by various stimuli such as breathing, temperature, body posture, emotion, or physical stress (40, 41). Studies have shown that blood pressure drops slowly and eventually slows down within 16 min of sitting in a chair before blood pressure measurements are taken, and 75% of the blood pressure drops occur in the first 10 min (42). In addition, heart rate (HR) is closely related to blood pressure. For patients with hypertension, the increase of HR further increases the risk of adverse outcomes. There is evidence that HR is an independent risk factor for cardiovascular disease morbidity and for the overall mortality in patients with hypertension (43). In this review, resting systolic blood pressure, resting diastolic blood pressure, and resting HR were selected as the main indicators to evaluate the condition of patients with hypertension, and BMI was selected as the simple and commonly used index to evaluate the degree of obesity.

To better understand the effect of exercise on the hypertension population, this review aims to make indirect and mixed comparisons of the interventions on rest SBP, rest DBP, rest HR, and BMI in patients with hypertension using a network meta-analysis method. So far, no studies have adjusted and mixed comparisons. Hence, we implemented the adjusted and mixed comparisons with network meta-analysis to provide better advice for the hypertension population.

## Method

This review was conducted based on the Preferred Reporting Items for Systematic Reviews and Meta-Analysis guidelines (PRISMA). Literature collection, exclusion criteria, and retrieval strategies are jointly proposed and agreed upon by two authors and established a priori to minimize bias.

### Data Acquisition

The study included randomized design studies published before August 2021. This study was reviewed by another peer. The population, interventions, comparisons, and outcomes of this review were as follows:

#### Participants/Population

The subjects in the study included were adults with high blood pressure. According to the hypertension guidelines of the American Heart Association, an SBP value between 130 and 139 mmHg and/or a DBP value between 80 and 89 mmHg is considered to be stage 1 arterial hypertension 43. In this review, 12 studies were included, with a total of 846 subjects aged between 31.8 and 78 years old.

#### Intervention(s)

The exercise interventions in this review include high-volume high-intensity interval training (HVHIIT), low-volume high-intensity interval training (LVHIIT), medium-intensity training (MIT), general physical activity, and blank. This review excluded studies that had only one intervention.

According to the classification of previous studies, in this review, the criteria for the variety of LVHIIT are as follows: (1) Exercise in which the heart rate is ≥80% maximum heart rate (max HR), and the total duration is not more than 30 min; (2) Exercise in which the oxygen uptake is ≥80% maximal oxygen consumption (Max VO2), and the total duration is not more than 30 min; (3) Exercise in which the power is ≥80% peak power, and the total duration does not exceed 30 min; and (4) The authors of the study classified it as LVHIIT. The high-volume high-intensity interval training (HVHIIT) classification criteria are as follows: (1) Exercise in which the heart rate is ≥80% maximum heart rate (max HR), and the total duration is more than 30 min; (2) Exercise in which the oxygen uptake is ≥80% maximum VO2, and the total duration is more than 30 min; (3) Exercise in which the power is ≥80% peak power, and the total duration is more than 30 min; and (4) The authors of the study classified it as HVHIIT. The medium intensity training (MIT) classification criteria are as follows: (1) Exercise with an average HR between 55 and 80% max HR; (2) Oxygen uptake during exercise is between 55 and 80% maximum VO2; (3) Exercise with an average power of 50–60% peak power; and (4) The authors of the study specified it as MIT.

In addition, among the 12 studies included, 9 used treadmills or power bicycles to test the maximum heart rate, maximum oxygen uptake, or maximum power (38, 39, 44–48) of the subjects, and 1 study used sub-maximum oxygen uptake to estimate maximum oxygen uptake (49), while the other two did not elaborate on it (50, 51).

#### Comparator(s)/Control

The indirect comparisons of the above interventions are feasible because the network meta-analysis is based upon the theorem of Bayes (51). The comparator(s)/control criteria were the same as the intervention(s) criteria.

#### Outcomes

The outcome indicators of this review are SBP, DBP, BMI, and rest HR.

Other indicators included in this review were too rare or had different detection methods to perform a reticular meta-analysis, so they were treated as secondary indicators for supplementary analysis. These indicators included the following: time to exhaustion, ventilatory thresholds, body fat, Max VO2, total cholesterol, max HR, and mean arterial pressure.

### Information Sources

This review uses PubMed, Medline, Cochrane Library, and CINAHL to conduct a comprehensive and repeatable literature search before August 2021. If the data is insufficient, the author can be contacted to provide the exact data.

### Search

(1) In PubMed, the search term was “(hypertension [Title/Abstract]) OR (blood pressure [Title/Abstract]) AND (HIIT [Title/Abstract]) OR (high intensity interval training [Title/Abstract]), OR (high intensity interval [Title/Abstract]), AND (randomized [Title/Abstract]) OR [(randomized [Title/Abstract]).”

(2) In Medline, Cochrane Library, and CINAHL, the search term was “(hypertension OR blood pressure TI) OR (hypertension OR blood pressure AB), AND (HIIT OR high-intensity interval training OR high-intensity interval TI), AND (randomized OR randomized AB).”

The selection of the title, abstract, and full text is jointly completed by two independent authors. The differences will be judged by a third independent arbitrator.

### Study Selection

The process of screening the abstract and the text is done by two independent authors. When no opinion can be reached, the disagreement will be judged by a third independent arbitrator.

Studies would be excluded if they meet the following conditions: (1) Studies with healthy subjects or minors; (2) Studies which only performed one-time exercise; (3) Studies using invasive interventions such as surgery and injections; and (4) Studies in which specific data of outcome indicators are not provided, or where the authors do not receive timely answers.

### Data Collection Process

All potential studies were downloaded and imported into Endnote X9 (Thomson Reuters, Carlsbad, California, USA), and the duplicated tasks were deleted. The data collection is done by two independent authors. When the opinions cannot be reached, the third independent arbitrator will judge the differences. The information included demographic characteristics (average age and gender), clinical characteristics (Body mass index), details of experimental design (sample size, intervention method, and follow-up time), and outcome indicators.

### Data Items

The funders of this study did not contribute to design or implementation. Therefore, the author is fully responsible for data collection, analysis, interpretation, and reporting. Corresponding authors have access to all data and are ultimately responsible for the submission of publications.

### Risk of Bias in Assessment

Two evaluators evaluate the risk of bias using the Cochrane Collaboration Risk of Bias Assessment Tool. When no agreement can be reached, the disagreement will be judged by a third independent arbitrator.

### Summary Measures

The data preprocessing and analysis were made by two independent investigators. Microsoft Excel (Version 16.0, Microsoft Corporation, Redmond, WA, USA) was used to pre-process the original data and convert the results into average and standard deviation (Mean ± SD).

The processed data are analyzed by the Aggregate Data Drug Information System (ADDIS V1.16.8 produced by Drugis.org, http://drugis.org/software/addis/index), calculated the effect size, the data are aggregated into the network meta-analysis, and all the graphs and results were the output. The results of the network meta-analysis are introduced in the following parts.

## Results

### Search Strategy and Information Extraction

A total of 1,580 studies were searched for screening through the electronic search of four scientific databases of which 310 repetitive studies were deleted; after filtering by title and abstract, additional 1,258 articles were excluded. Finally, 12 studies with all subjects age between 18 and 78; and all these subjects included in the analysis were patients that were hypertensive, with an SBP value of more than 130 mmHg and/or a DBP value of more than 80 mmHg.

**Figure 1:**
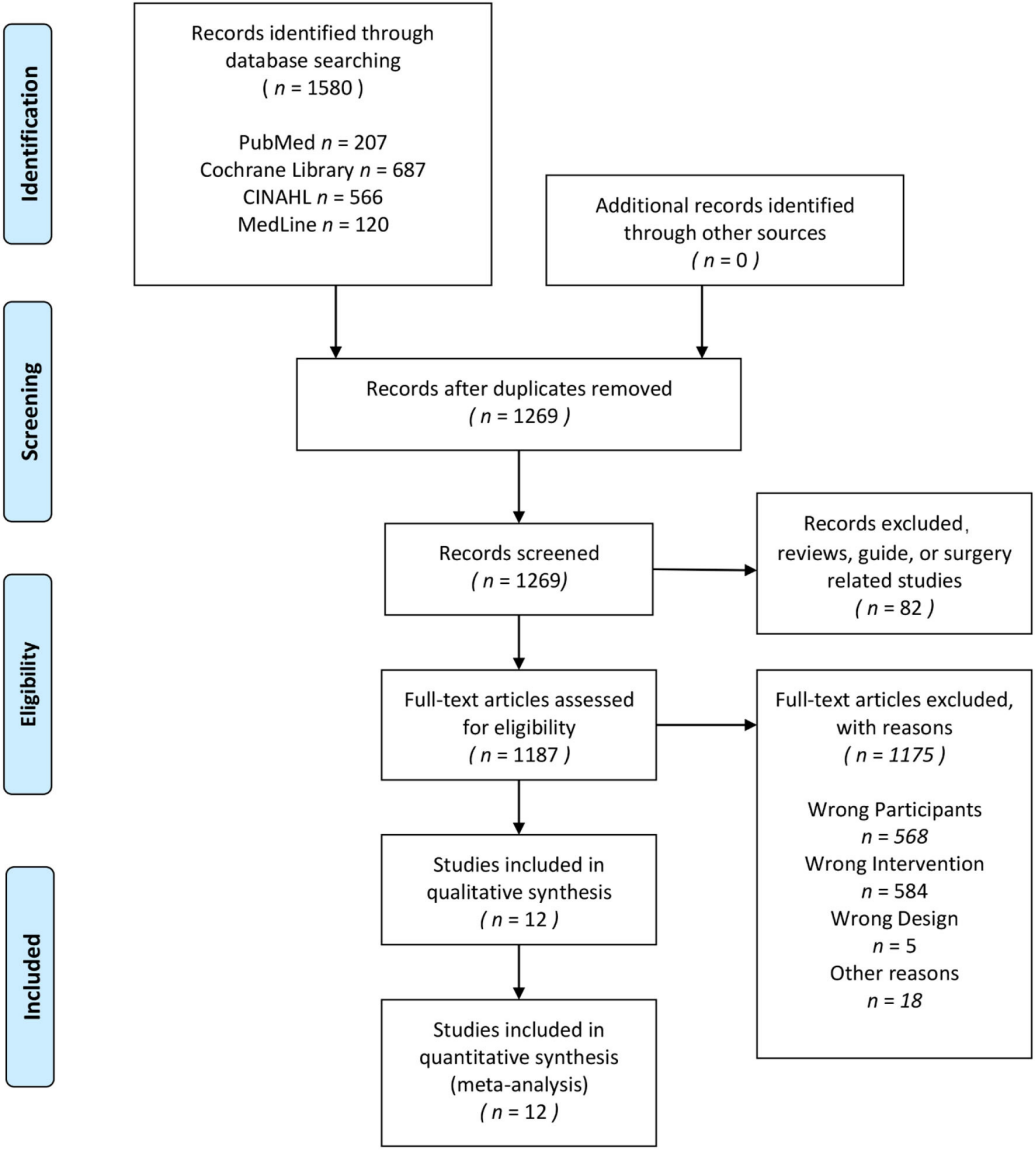
PRISMA flow diagram for the systematic review and the network meta-analysis. Adapted from Moher et al. (52). For more information, visit www.prisma-statement.org.

Figure 1 and Table 1 show the details of the article filtering process and the information of all included studies, respectively.

**Table 1:** The study characteristics of included studies.

**References**	**Title**	**Subjects**			**Intervention**			**Index**
		**Average age (n-n years)**	**Male/ Female**	**N**	**Type of training**	**Training frequency**	**Content**	
Dalal et al. (43)	Short-duration high-intensity interval exercise training is more effective than long duration for blood pressure and arterial stiffness but not for inflammatory markers and lipid profiles in patients with stage 1 hypertension	48.0 (43.0–53.8)	30/0	10	LVHIIT	3 times/week, 8 weeks	80%VO2max 27 × 30 s	SBP (↓), DBP (↓)
				10	LVHIIT		85%VO2max 4 × 4 min	SBP (↓), DBP (↓)
				10	Blank		Blank	SBP (↔), DBP (↔)
Bahmanbeglou et al. (44)	The benefits of high-intensity interval training on cognition and blood pressure in older adults with hypertension and subjective cognitive decline: results from the heart & mind study	71.1 (63.3–78.0)	67/61	65	LVHIIT	3 times/week, 24 weeks	85–95% HRmax 25 min	SBP (↓), Time to exhaustion (↑)
				63	MIT		60–80% HRmax 25 min	SBP (↓), Time to exhaustion (↑)
Lins-Filho et al. (49)	Effects of interval training on blood pressure and endothelial function in hypertensive patients	51.3 (40.9–61.1)	8/6	7	LVHIIT	5 times/week, 4 weeks	80% HRmax 5 × 3 min	SBP (↓), DBP (↓), BMI (↔), Rest HR (↓)
				7	MIT		60% HRmax 35 min	SBP (↓), DBP (↔), BMI (↔), Rest HR (↔)
Whitaker et al. (37)	Effects of different aerobic exercise programs with nutritional intervention in sedentary adults with overweight/obesity and hypertension: EXERDIET-HTA study	54.0 (44.4–63.5)	120/55	40	MIT	2 times/week, 16 weeks	65% VO2max 45 min	SBP (↓), DBP (↓), BMI (↓), Rest HR (↓), MBP (↓), Anaerobic thresholds (↔)
				42	HVHIIT		95% VO2max 45 min	SBP (↓), DBP (↓), BMI (↓), Rest HR (↓), MBP (↓), Anaerobic thresholds (↑)
				41	LVHIIT		90% VO2max 20 min	SBP (↓), DBP (↓), BMI (↓), Rest HR (↓), MBP (↓), Anaerobic thresholds (↑)
				40	General physical activity		General physical activity	SBP (↓), DBP (↓), BMI (↓), Rest HR (↓), MBP (↓), Anaerobic thresholds (↔)
Boa Sorte Silva et al. (45)	High-intensity interval training lowers blood pressure and improves apelin and NOx plasma levels in older treated hypertensive individuals	61.7 (51.1–69.3)	23/19	Unknown	LVHIIT	3 times/week, 6 weeks	85–90% HRmax 35 min	SBP (↓), DBP (↓), Rest HR (↔), Max VO2 (↑), Time to exhaustion (↑)
				Unknown	Blank		blank	SBP (↔), DBP (↔), Rest HR (↔), Max VO2 (↔), Time to exhaustion (↔)
Izadi et al. (46)	Effects of high-intensity interval training vs. moderate-intensity continuous training on epicardial fat thickness and endothelial function in hypertensive metabolic syndrome	50.9 (42.6–60.3)	18/16	17	LVHIIT	3 times/week, 8 weeks	85% HRmax 5 × 3 min	SBP (↓), DBP (↓), BMI (↓), Rest HR (↓), Total cholesterol (↓)
				17	MIT		60 HRmax 35 min	SBP (↓), DBP (↓), BMI (↓), Rest HR (↔), Total cholesterol (↔)
Sosner et al. (48)	Affective responses to different prescriptions of high-intensity interval exercise in hypertensive patients	65.3 (61.1–69.5)	0/20	Unknown	LVHIIT	8 times	80–85% VO2max 5 × 2 min	SBP (↔), DBP (↔), Rest HR (↔)
				Unknown	Blank		blank	SBP (↔), DBP (↔), Rest HR (↓)
Gorostegi-Anduaga et al. (38)	Effects of different aerobic exercise programs on cardiac autonomic modulation and hemodynamics in hypertension: data from EXERDIET-HTA randomized trial	53.7 (31.8–61.7)	158/91	61	HVHIIT	16 weeks	45 min	SBP (↓), DBP (↓), Rest HR (↓), Max VO2 (↑)
				62	LVHIIT		20 min	SBP (↓), DBP (↓), Rest HR (↓), Max VO2 (↑)
				60	MIT		45 min	SBP (↓), DBP (↓), Rest HR (↓), Max VO2 (↑)
				59	General physical activity		General physical activity	SBP (↓), DBP (↓), Rest HR (↓), Max VO2 (↔)
Soltani et al. (53)	Effects of antihypertensive medication and high-intensity interval training in hypertensive metabolic syndrome individuals	58.7 (53.2–64.2)	Unknown	Unknown	LVHIIT (Take Placebo)	3 times/week, 16 weeks	90% HRmax 4 × 4 min/5 × 5 min (Take Placebo)	SBP (↔), DBP (↔), Rest HR (↔), Mean arterial pressure (↔)
				Unknown	LVHIIT (Take antihypertensive drug)		90% HRmax 4 × 4 min/5 × 5 min (Take antihypertensive drug)	SBP (↔), DBP (↔), Rest HR (↓), Mean arterial pressure (↔)
Taha et al. (51)	High-intensity interval training irrespective of its intensity improves markers of blood fluidity in hypertensive patients	48.0 (43.0–53.8)	30/0	10	LVHIIT	3 times/week, 8 weeks	80–100%VO2max 2 × 30 s	SBP (↓), MAP (↔), BMI (↔)
				10	LVHIIT		75–90%VO2max 4 × 4 min	SBP (↓), MAP (↓), BMI (↔)
				10	Blank		Blank	SBP (↔), MAP (↔), BMI (↔)
Jo et al. (47)	Ambulatory blood pressure reduction following 2 weeks of high-intensity interval training on an immersed ergo cycle	65.0 (54.0–72.0)	22/20	Unknown	LVHIIT	3 times/week, 2 weeks	100% Peak Power 2 × 15 s (Dryland)	SBP (↔), BMI (↔), Rest HR (↔)
				Unknown	LVHIIT		100% Peak Power 2 × 15 s (Immersed)	SBP (↓), BMI (↓), Rest HR (↓)
				Unknown	MIT		50%PeakPower	SBP (↔), BMI (↔), Rest HR (↔)
Jo et al. (50)	Effect of high-intensity interval training on endothelial function in postmenopausal hypertensive patients: a randomized controlled trial	48.0 (45.2–50.4)	0/46	23	LVHIIT	3 times/week, 10 weeks	80–85% HRmax 4 × 4 min	SBP (↓), DBP (↓), BMI (↔)
				23	Blank		Blank	SBP (↔), DBP (↔), BMI (↔)

*Blank, Blank control; LVHIIT, low-volume high-intensity interval training; HVHIIT, high-volume high-intensity interval training; MIT, Medium intensity training; SBP, systolic blood pressure; DBP, diastolic blood pressure; BMI, body mass index; HR, heart rate; Max VO2, maximal oxygen consumption; ↑, Significantly rise; ↓, Significantly decrease; ↔, No statistically significant change*.

### Risk of Bias

**Figure 2:**
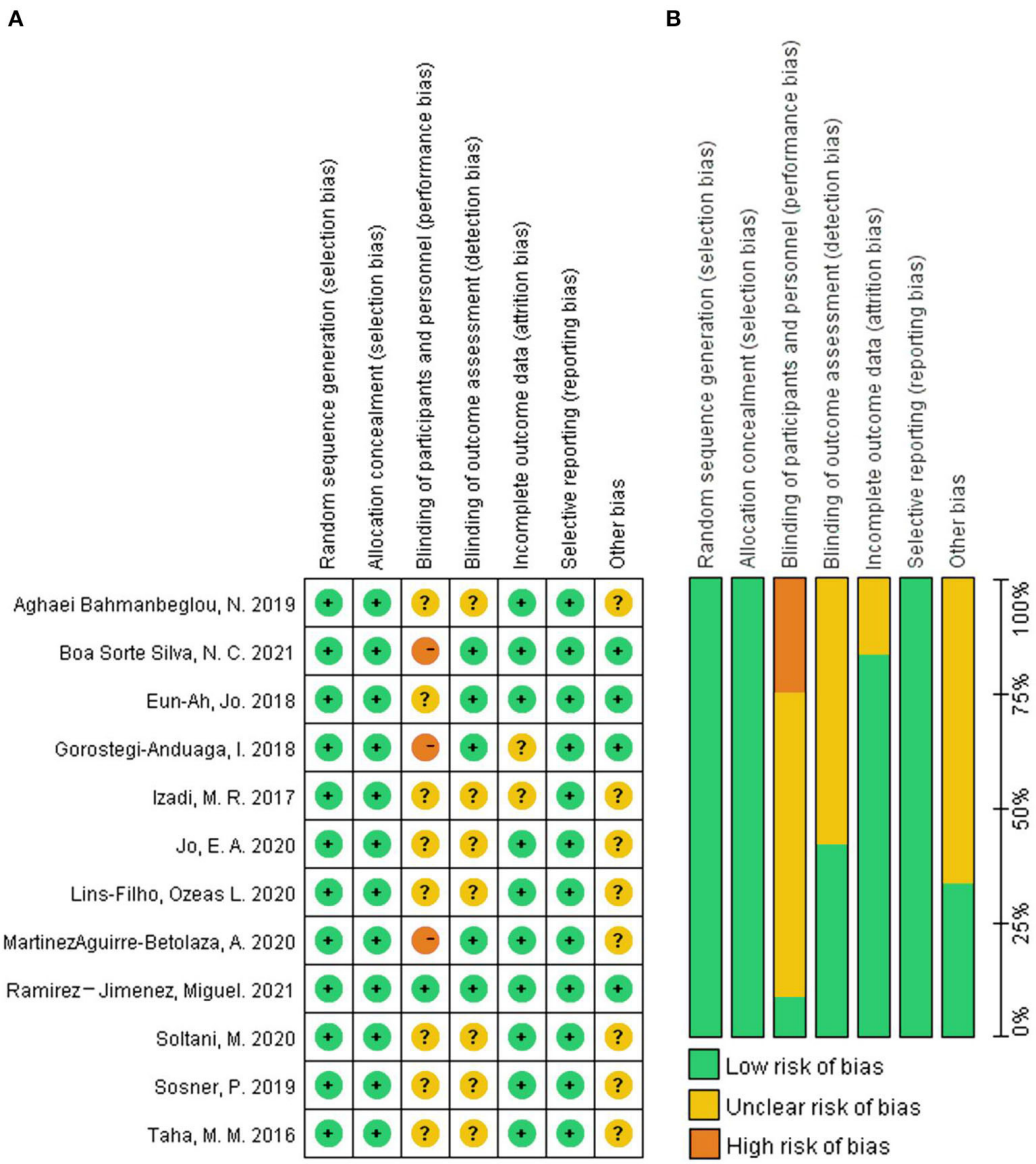
The result of the risk of bias assessment. **(A)** Risk of bias graph; **(B)** Risk of bis summary.

The risk of bias in the 12 included studies was assessed, and the consensus was reached after discussion. The overall result is shown in Figure 2. Randomization and concealment methods of the participants were well-reported in all studies. In percentage, 66.7% of studies did not adequately describe participant or staff blinding, and 25% of the studies made it clear that there was no double blindness. Proportionately, 58.3% of the studies did not describe whether the evaluator was blind. Two studies had incomplete results due to subjects dropping out. All the studies recorded their research plan and researched according to the program.

### Network Meta-Analysis

**Figure 3:**
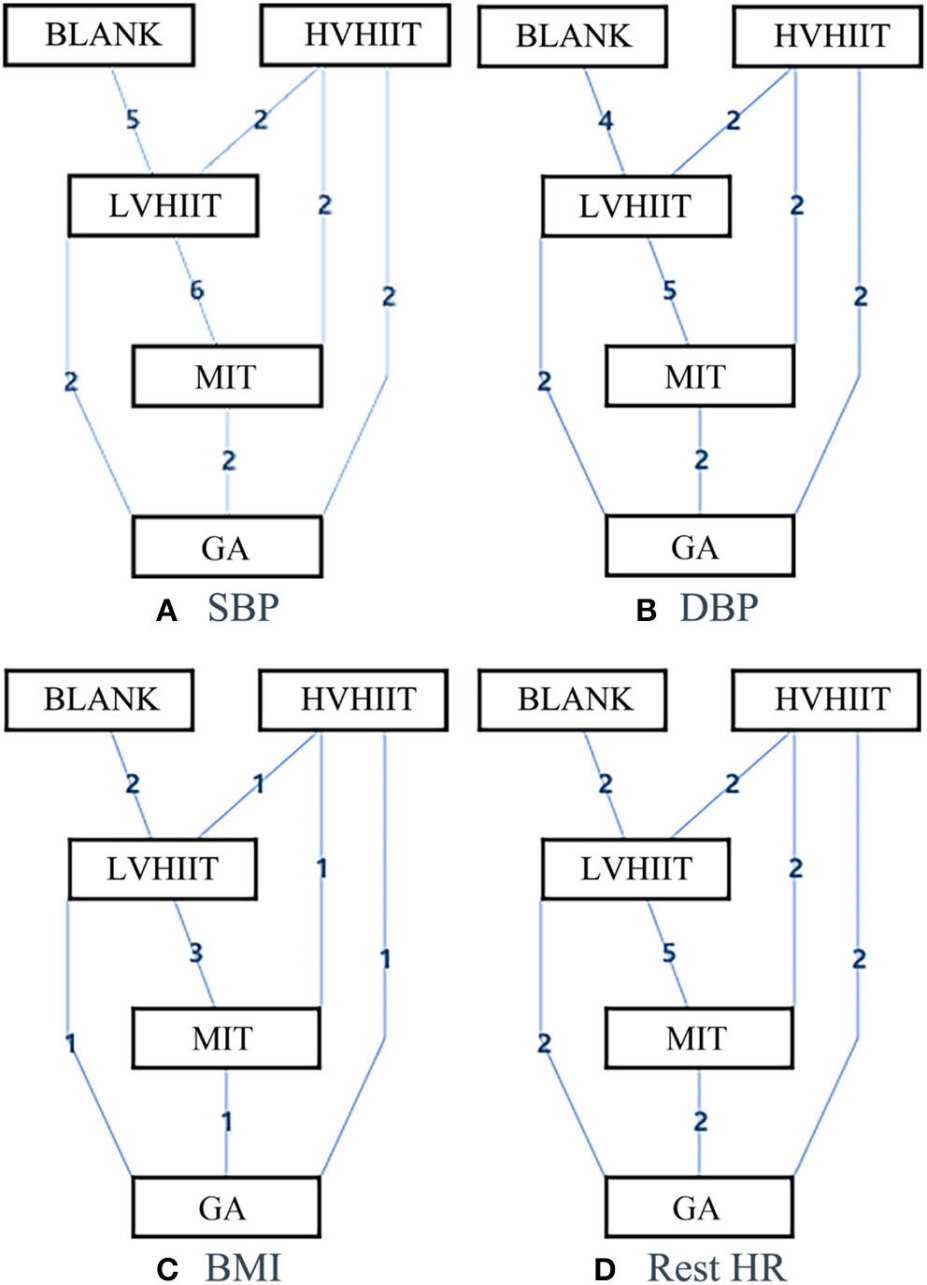
The network structure of the intervention of systolic blood pressure (SBP), diastolic blood pressure (DBP), body mass index (BMI), and rest heart rate (HR). Blank, Blank control; LVHIIT, low-volume high-intensity interval training; HVHIIT, high-volume high-intensity interval training; MIT, Medium intensity training; GA, general physical activity.

**Figure 4:**
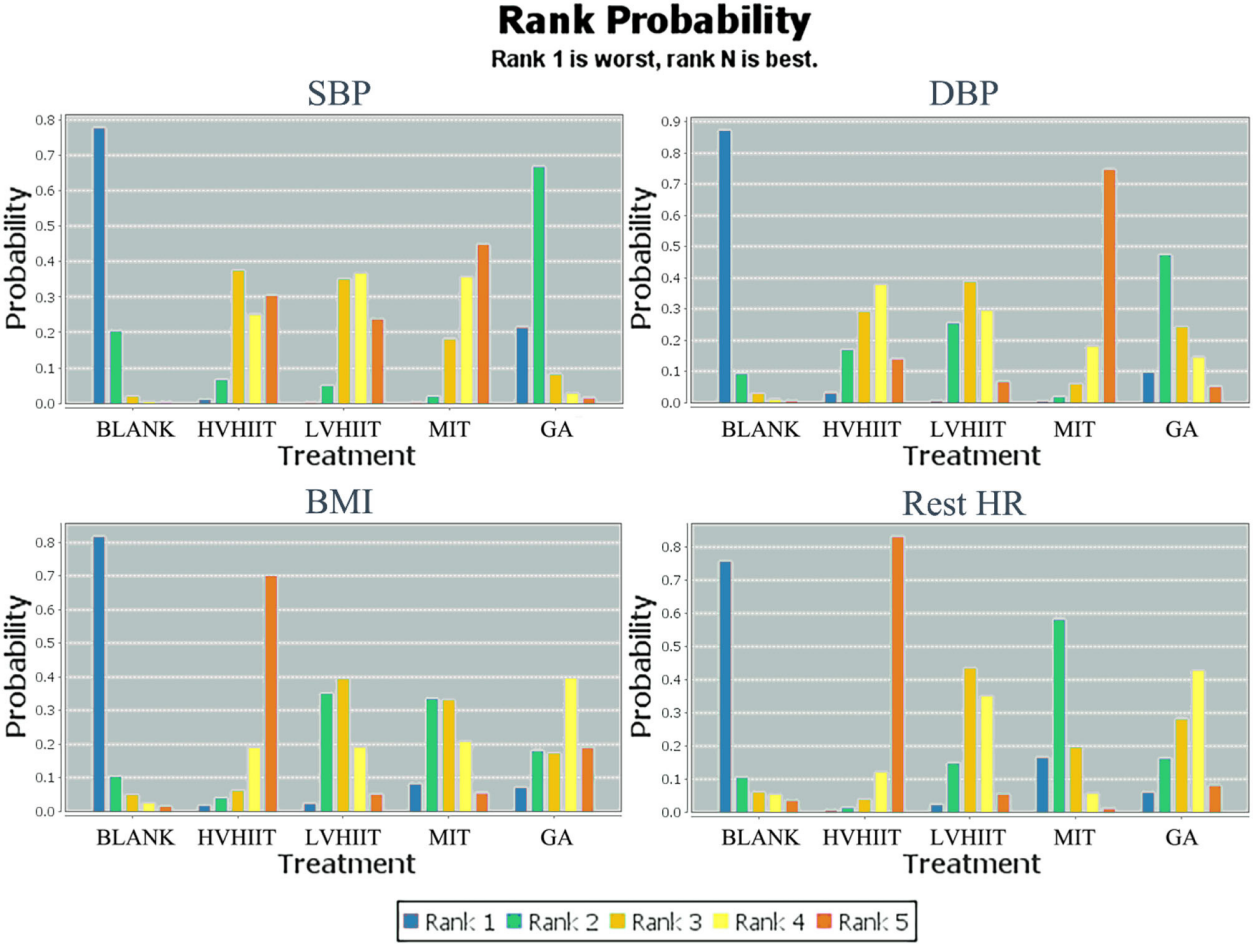
Measurement of systolic blood pressure (SBP), diastolic blood pressure (DBP), body mass index (BMI), rest heart rate (HR), and the ranking of intervention probability. Blank, Blank control; LVHIIT, low-volume high-intensity interval training; HVHIIT, high-volume high-intensity interval training; MIT, Medium intensity training; GA, general physical activity.

Figures 3, 4 show the overall network structure of intervention for systolic blood pressure (SBP), diastolic blood pressure (DBP), body mass index (BMI), and rest heart rate (RHR) with mixed intervention comparison of various training activities, and their respective ranking of intervention probability by ranking 1 as the worst, and ranking 5 as the best, respectively.

#### Systolic Blood Pressure

Figure 3A shows the geometry of the SBP motion intervention network. It shows a mixed intervention comparison that developed from the traditional Meta-analysis. It expanded from the standard double-arm test Meta-analysis to a series of different treatment factors to analyze, compare each other and synthesize simultaneously. The mixed intervention comparison includes direct comparison and indirect comparison. As the evidence is a closed loop, the inconsistency of the evidence was evaluated.

In the mixed comparison of HVHIIT, LVHIIT, MIT, blank, and general physical activity, the random effect standard deviation of the consistency model showed 95% confidence interval of 3.18 (1.52, 6.39), while the random effect standard deviation of the inconsistency model showed 95% confidence interval of 3.22 (1.51, 6.51). The standard inconsistency deviation of the inconsistency model with 95% confidence interval was 5.90 (0.30, 11.61). There were no significant differences in the standard deviations of random effects between the congruent and incongruent models, suggesting there is no consistency difference. Hence, the consistency model should be used. Table 2 shows the interventions for systolic blood pressure (SBP) based on the network geometry of Figure 3A.

**Table 2:** The league table of the interventions for systolic blood pressure (SBP).

**Blank**				
7.21 (0.14, 14.41)	HVHIIT			
7.27 (2.51, 11.53)	0.04 (−5.88, 5.26)	LVHIIT		
7.96 (2.25, 14.09)	0.77 (−4.63, 6.19)	0.69 (−2.86, 5.03)	MIT	
2.78 (−4.49, 10.06)	−4.44 (−10.95, 1.90)	−4.49 (−10.34, 1.71)	−5.20 (−11.44, 0.60)	General physical activity

*Blank, Blank control; LVHIIT, low-volume high-intensity interval training; HVHIIT, high-volume high-intensity interval training; MIT, Medium intensity training*.

From Figure 4, the results show the lower the SBP, the better the situation. So, for patients with hypertension, the best to worst means of reducing SBP are MIT, LVHIIT, HVHIIT, general physical activity (GA), and blank.

#### Diastolic Blood Pressure

Figure 3B shows the geometry of the DBP motion intervention network with a mixed intervention comparison. The mixed intervention comparison includes direct comparison and indirect comparison. Similarly, as the evidence is a closed loop, the inconsistency of the evidence should be evaluated.

In the mixed comparison of HVHIIT, LVHIIT, MIT, blank, and general physical activity, the random effect standard deviation of the consistency model showed 95% confidence interval of 1.54 (0.17, 3.99), while the random effect standard deviation of the inconsistency model showed 95% confidence interval of 1.55 (0.28, 3.99). The standard inconsistency deviation of the inconsistency model with 95% confidence interval was 3.39 (0.16, 6.60). There were no significant differences in the standard deviations of random effects between the congruent and incongruent models, hence, the consistency model was adopted. Table 3 shows the intervention for systolic blood pressure (DBP) based on is a sorted table of the network geometry of Figure 3B.

**Table 3:** The league table of the interventions for diastolic blood pressure (DBP).

**Blank**				
3.72 (−0.88, 7.67)	HVHIIT			
3.41 (0.40, 5.99)	−0.32 (−3.43, 2.95)	LVHIIT		
5.04 (0.94, 8.77)	1.37 (−1.75, 4.60)	1.66 (−1.15, 4.35)	MIT	
2.65 (−1.93, 6.86)	−0.97 (−4.51, 2.56)	−0.68 (−4.17, 2.69)	−2.35 (−5.75, 1.03)	General physical activity

*Blank, Blank control; LVHIIT, low-volume high-intensity interval training; HVHIIT, high-volume high-intensity interval training; MIT, Medium intensity training*.

Figure 4 shows the ranking of measurements and of probabilities. Noting that the lower the DBP in this study, the better the situation, thus, the best-to-worst ways to reduce DBP for patients with hypertension are MIT, HVHIIT, LVHIIT, general physical activity (GA), and blank.

#### Body Mass Index

Similarly, Figure 3C shows the geometry of the BMI motion intervention network with a mixed intervention comparison. The mixed intervention comparison includes direct comparison and indirect comparison. As there is evidence of a closed loop, the inconsistency of the evidence was evaluated.

In the mixed comparison of HVHIIT, LVHIIT, MIT, blank, and general physical activity, the random effect standard deviation of the consistency model showed a 95% confidence interval of 0.72 (0.02, 1.82), the random effect standard deviation of the inconsistency model showed 95% confidence interval of 0.71 (0.04, 1.82). The standard inconsistency deviation of the inconsistency model with 95% confidence interval was 0.96 (0.05, 1.88). There were no significant differences in the standard deviations of random effects between the congruent and incongruent models, indicating no consistency difference, and the consistency model should be adopted. Table 4 showed the interventions for BMI based on the network geometry of Figure 3C.

**Table 4:** The league table of the interventions for body mass index (BMI).

**Blank**				
2.76 (−0.42, 5.41)	HVHIIT			
1.40 (−0.52, 2.90)	−1.37 (−3.62, 1.00)	LVHIIT		
1.32 (−1.16, 3.48)	−1.38 (−3.70, 0.98)	−0.02 (−1.63, 1.60)	MIT	
1.83 (−1.32, 4.54)	−0.93 (−3.40, 1.59)	0.47 (−2.02, 2.86)	0.48 (−1.97, 2.88)	General physical activity

*Blank, Blank control; LVHIIT, low-volume high-intensity interval training; HVHIIT, high-volume high-intensity interval training; MIT, Medium intensity training*.

Figure 4 shows the ranking of measurements and of probabilities. Noting that the lower the BMI in this study, the better the situation, thus, the best-to-worst ways to reduce BMI for patients with hypertension is HVHIIT, GA, LVHIIT, MIT, and blank. It is important to note that LVHIIT and MIT are very close in reducing the effectiveness of BMI.

#### Rest Heart Rate

Figure 3D shows the geometry of the rest HR motion intervention network with a mixed intervention comparison. As there is evidence of a closed loop for direct comparison and indirect comparison, the inconsistency of the evidence was again evaluated.

In the mixed comparison of HVHIIT, LVHIIT, MIT, blank, and GA, the random effect standard deviation of the consistency model showed 95% confidence interval of 1.17 (0.11, 4.75), the random effect standard deviation of the inconsistency model showed 95% confidence interval of 1.13 (0.09, 4.80). The standard inconsistency deviation of the inconsistency model with 95% confidence interval was 4.07 (0.20, 7.91). There were no significant differences in the standard deviations of random effects between the congruent and incongruent models, indicating no consistency difference, hence, the consistency model was used. Table 5 showed the interventions for HR based on the network geometry of Figure 3D.

**Table 5:** The league table of the interventions for rest heart rate (HR).

**Blank**				
6.05 (−0.88, 12.57)	HVHIIT			
3.60 (−2.87, 9.35)	−2.36 (−5.79, 1.21)	LVHIIT		
2.49 (−3.84, 8.53)	−3.51 (−6.83, −0.06)	−1.23 (−3.98, 1.68)	MIT	
3.90 (−3.12, 10.35)	−2.14 (−5.82, 1.44)	0.17 (−3.45, 3.89)	1.47 (−2.25, 4.81)	General physical activity

*Blank, Blank control; LVHIIT, low-volume high-intensity interval training; HVHIIT, high-volume high-intensity interval training; MIT, Medium intensity training*.

In the ranking of measurements and probabilities, as shown in Figure 4, with the lower rest HR as the better situation, the results show that for patients with hypertension, HVHIIT, GA, LVHIIT, MIT, and blank are the means to reduce the rest HR from the best to the worst.

## Discussion

In this review, the network meta-analysis method is used to mix and indirectly compare HIIT with different volumes and other types of sports. This study aimed to determine the effects of different volumes and types of exercise on the decrease of blood pressure and other health conditions in people with hypertension. According to the exercise intervention classification, 12 randomized controlled trials of five different interventions: HVHIIT, LVHIIT, MIT, general physical activity (GA), and blank were reviewed. All the subjects studied were people with hypertensive and with SBP >130 mmHg and/or DBP >80 mmHg.

All of the 12 studies reported on SBP and 11 of them showed that SBP levels decreased significantly compared with the baseline, after a period of exercise training (*p* < 0.05) (38, 39, 44–51, 53), only 1 study showed no significant change in SBP levels after exercise (*p* > 0.05) (54). Of the 9 studies reported on DBP (38, 39, 44, 46, 47, 49–51, 54), only 1 study showed no significant change in DBP after exercise (*p* > 0.05) (49), and the other 8 studies showed DBP decreased significantly after the exercise. Subsequently, 6 studies reported on BMI (38, 47, 48, 50, 51, 53), and only 2 studies showed a significant decrease in BMI after exercise (38, 47, 48). Based on 8 studies reported on rest HR (38, 39, 46–50, 54), 6 studies showed rest HR decreased after exercise (38, 39, 47, 48, 50, 54), and 2 studies showed rest HR did not change significantly after exercise (46, 49). However, in the study by Lins-Filho et al., rest HR decreased significantly in the blank control group (49).

The review shows that proper physical exercise is beneficial to hypertension patients with stable health. In the 12 studies included, almost all the different volumes and types of training positively impacted the health status of patients with hypertension, and no adverse effects of exercise as an intervention were reported. And some studies have shown the exercise ability of the subject improved to a certain extent after receiving exercise intervention for a period of time (38, 45, 46).

According to our analysis, MIT is the best in reducing SBP, while the blank is the worst, suggesting exercise training can improve SBP in people with hypertension, which is agreed with the results of many previous studies (55–57). The effect of high-volume HIIT or low-volume HIIT on reducing SBP seems to be less than that of MIT, which may be related to the mechanism of lowering blood pressure on these two types of exercise. The antihypertensive effect of HIIT may be caused by multiple factors, such as the decrease of cardiac output, heart rate, and vascular resistance (55).

For DBP, MIT is still the best, and blank is the worst in reducing DBP. The effect of HIIT with different capacities on reducing DBP is similar, and it is not as good as MIT. In reducing BMI, the effect of HVHIIT is the best, and the list is blank.

In terms of reducing rest HR, HVHIIT is the best, and blank is the worst. A study by Lins-Filho et al. (49), reported that SBP did not change significantly in HIIT and blank groups, but with a slight increase in SBP when the measurement time was taken 60 min after exercise. The transient increase in blood pressure from the exercise did not fully recover after rest. In Ramirez-Jimenez et al. (54) investigating of both groups of subjects received LVHIIT intervention, with a group taking antihypertensive drugs and the other group receiving placebo, aiming to achieve an additional drop in blood pressure. However, the results showed only the placebo group had a significant decrease in resting heart rate (*p* < 0.05), and no statistically significant difference was found in other indicators. We hypothesize that MIT seems to have a significant advantage in reducing blood pressure, while HVHIIT is effective in reducing BMI and rest HR.

Of the 12 studies included, only Martinez Aguirre-Betolaza et al. gave follow-up results (39). In this study, long-term follow-up showed that the daytime DBP of the exercise group was lower than that of the subjects with general physical activity, and the blood pressure variability (quantified as 24 h, day, and night average SD) of the exercise intervention subjects was <16 mmHg, while the nocturnal blood pressure variability of the subjects with general physical activity was greater than that of 16 mmHg. Hence, exercise intervention seems to reduce the occurrence of cardiovascular risk events.

Though there is evidence showing that exercise has a positive effect on blood pressure and health in people with high blood pressure, excessive strenuous exercise in the short term may increase the risk (36). Therefore, it is necessary to recommend appropriate exercise according to the severity of hypertension. In addition, the effects of different types and intensity of exercise on different subjects may be different. A study by Danielle et al., on the effects of age and gender on resting blood pressure changes caused by grip strength training, showed older women had the most significant decrease in SBP (58); patients who are obese and hypertensive showed improved insulin resistance during exercise-induced fat loss (59); exercise as an auxiliary means of lowering blood pressure combined with taking antihypertensive drugs seemed to achieve better blood pressure control (60). In the following sections, we further explore and discuss these various effects.

### Age

It is evident that exercise improves blood pressure, but the effect may vary according to age. Compared with young patients with hypertension, elderly and frail patients are more likely to develop age-related diseases, and their adverse side effects and adverse results are often more worrying (61).

According to the definition of age defined by the United Nations World Health Organization, of the 12 studies evaluated, 8 studies were young hypertensive patients aged 31.8–63.5 years old (38, 39, 44, 47, 50, 51, 53, 54), and 7 of these studies showed that after a period of physical exercise, the blood pressure and/or other health conditions of the subjects improved significantly (*p* < 0.05). However, there was no statistically significant change in SBP and DBP after receiving HIIT exercise intervention (54). All the subjects who participated in HIIT exercise intervention were patients with metabolic syndrome in addition to high blood pressure, with one group of subjects also receiving antihypertensive drugs during the intervention, while the other group was receiving a placebo (54). Therefore, the insignificant change in blood pressure may be due to the heterogeneity of different diseases or the side effects of drug use.

In 3 of the four studies in subjects aged from 51.08 to 78 years old, their blood pressure and/or health status improved significantly after a period of physical exercise (45, 46, 48), and only one study had no statistically significant change in blood pressure after receiving HIIT intervention (49). For the study with subjects who received only eight HIIT interventions, the blood pressure was measured 60 min after each intervention, the insignificant change may be due to the short cycle of the intervention, whereby the blood pressure did not return to the resting level after exercise.

In the 12 studies that were analyzed, there was no difference in the improvement of blood pressure by exercise in different age groups of adult patients with hypertension. However, none of these studies are directly age-related, it is impossible to make a more accurate analysis of this topic. Further related studies needed to be carried out.

### Gender

Of the 12 studies included, only 4 included patients with hypertension of a single sex (44, 49, 51, 53). In the Bahmanbeglou et al. (44) and Soltani et al. (53) study, a total of 60 male patients with hypertension received HVHIIT, LVHIIT, and blank control intervention, respectively, significant improvement in blood pressure was observed in 40 subjects who received exercise intervention. In the Taha MM study (51), 46 female patients with hypertension received LVHIIT and blank control intervention, respectively. After 10 weeks of intervention with 80–85% max HR intensity HIIT, the blood pressure of the patients was significantly improved (*p* < 0.05). In Lins-Filho OL's study (49), it only reported that the resting heart rate of the blank control group has decreased significantly, which may be because the subjects in the exercise group received only 8 times interventions, with short rest time after exercise that their blood pressure levels had not fully recovered yet.

According to our results, the improvement of the blood pressure of male subjects after HIIT intervention is better than that of women, contrary to the results of previous studies (58). However, in the study of Danielle CB, subjects received grip resistance training, and different types of exercise led to different effects. In addition, the other 8 studies we revived did not indicate gender differences, and the relevant evidence is still too little to draw accurate conclusions on gender-related effects. More studies on the effects of exercise on patients with hypertension of different genders should be carried out in the future.

### Obesity

Of the 12 studies reviewed, only 11 studies reported the BMI levels of the subjects (38, 39, 45, 48, 49, 51, 53, 54). According to the BMI established by the World Health Organization (Between 25 and 29.9 is overweight, and more than 30 is obese), the patients were divided into standard weight (47, 50), overweight (45, 46, 48, 49, 53) or obese (38, 39, 51, 54) hypertensive ones according to the BMI index.

In the study of Jo et al. (47, 50), 48 subjects of standard weight received LVHIIT and MIT intervention, respectively. The results showed that the blood pressure levels of these subjects who received exercise intervention were significantly improved (*p* < 0.05), and the resting heart rate of subjects who received HIIT intervention also decreased significantly (*p* < 0.05).

Of the five studies with subjects who had overweight hypertension, only 2 had no significant blood pressure and health status changes after exercise intervention (48, 49). In the study of Sosner et al. (48) and Lins-Filho et al. (49), there were only 8 and 6 interventions in total, so the reason for this insignificance may be due to the short intervention cycle. In several other studies (45, 46, 53), 200 patients who are overweight and hypertensive showed significant improvements in blood pressure and/or other health conditions after exercise intervention (*p* < 0.05).

In 4 studies of patients that are obese with hypertension (38, 39, 51, 54), 3 studies reported significantly improved in blood pressure after exercise intervention (*p* < 0.05) (38, 39, 51), while Ramirez-Jimenez M study (54) did not show any significant change, which may be due to the effects of metabolic syndrome and additional antihypertensive drugs.

Our results showed no differences in blood pressure, weight loss, or other indicators in patients with hypertension with different body weights. However, in this review, there are only two studies in which the subjects are non-overweight people with BMI close to 25, it is impossible to make an accurate analysis of obesity-related issues in patients with hypertension, further studies are needed.

### Exercise

Neural activity may also affect cerebral blood flow during the exercise (62). Previous studies have shown that exercise training can reduce sympathetic excitation by reducing the activation of neurons in the cardiovascular region of the brain, thereby reducing the risk of cardiovascular disease (63). In addition, endurance training seems to reduce the intake of the brain of non-oxidizing carbohydrates and maintain brain oxygenation during sub-extreme exercise (62). However, in the 12 pieces of literatures included in this study, there is no mention of the movement of the sympathetic nervous system, and the effect of exercise on the activity of the sympathetic nervous system in people with hypertension needs to be further studied. Insulin resistance is closely related to arterial hypertension (64). Studies by Jelleyman et al. have shown that HIIT seems to improve metabolic health more effectively (65). However, studies on the effects of different intensity, volume, and types of exercise on patients with hypertension are still rare. Our previous studies have analyzed the effects of exercise on oxidative stress, and the results show that long-term high-intensity aerobic training has a better effect on the improvement of oxidative stress in people with chronic diseases (36). However, the effect of medium-intensity exercise on the improvement of oxidative stress is still controversial. A recent study showed that traditional Chinese exercise can also effectively improve the quality of life in patients with hypertension (66), however, no study has compared the effects of HIIT and traditional Chinese exercise on blood pressure control in patients with hypertension. However, for people with chronic diseases, exercise training programs are usually personalized to achieve better health results. Likewise, the effect of training is determined by many factors, including training time, intensity, interval times and types, etc. (67); thus, it is necessary to classify and analyze.

#### Volume

Of the 12 studies that were included, subjects in 4 studies performed HIIT or MIT for more than 30 min (38, 39, 47, 50). The results showed that the blood pressure levels of the subjects who received higher-volume exercise have improved significantly.

All 12 studies involved in low-volume exercise showed improvement in blood pressure, health, and exercise ability. On the contrary, Sosner et al. (48) and Ramirez-Jimenez et al. (54) studied with subjects who received 8 and 6 exercise interventions, respectively, and their blood pressure did not change significantly.

Generally, high-volume exercise has a good effect on improving the health status of patients with hypertension. However, only four studies on high-volume training are included in this review, lacking studies on the effects of different volume training, especially HIIT, on patients with hypertension. More evidence is needed to justify this result.

#### Intensity

For a long time, high-intensity exercise has been considered to produce more robust physiological adaptability (55). However, it has been reported that high-intensity aerobic exercise does not produce a better antihypertensive effect (23).

In the 12 studies included in this review, all groups that used HIIT as an intervention reported a significant improvement in blood pressure levels, and health indicators also showed a good development trend (38, 39, 44–47, 50, 51, 53). On the contrary, the study by Lins-Filho et al. (49) did not show a significant change in blood pressure after eight times of the HIIT intervention which may be due to either the short training period or the subjects had not fully recovered when the indicators were measured. In the study of Ramirez-Jimenez et al. (54), the subjects received HIIT intervention for 16 weeks, and their blood pressure levels did not change significantly, these subjects had metabolic syndrome or took additional medication antihypertensive drugs. In Sosner P's study (48), the blood pressure of the subjects did not change significantly after receiving HIIT intervention for a total of 6 times in 2 weeks. These subjects exercised twice at the intensity of 100% peak power for 15 s, except for warm-up and rest, which may lead to a low volume of the exercise cycle.

For 5 studies using MIT intervention (38, 39, 45, 47, 50), the blood pressure levels of the subjects showed significant improvement.

In 2 control group studies, the subjects followed the physical activity guidelines for low-intensity general physical activity. The results showed that the blood pressure levels, and resting heart rate of these subjects were significantly improved (*p* < 0.05).

According to our results, MIT seems to be the best way to improve hypertension in patients. However, it is worth noting that HIIT is more effective than MIT in reducing BMI and resting HR in patients, and in the studies of Gorostegi-Anduaga et al. (38), Boa Sorte Silva et al. (45), and Izadi et al. (46), the exercise ability of the subjects was significantly improved after the intervention of HIIT, which was characterized by a longer time to reach exhaustion or an increase in anaerobic threshold.

### Limitations

Due to a high degree of clinical heterogeneity and lack of data, our analysis cannot extrapolate to all the movements. However, similar trends in blood pressure and other health indicators were observed in patients with hypertension who received exercise intervention.

The limitations of this study are as follows: (1) The number of articles included in the reticular meta-analysis is limited, and the publication bias cannot be evaluated; (2) The patients with hypertension included in the study may also take antihypertensive drugs in addition to exercise intervention, but they are rarely mentioned in the articles; (3) Insufficient sample size may lead to overestimation of the intervention effect; (4) The mechanism of blood pressure reduction is a complex process, and our reticular meta-analysis is only a simplified method for this phenomenon; (5) In recent years, there are few studies on the effects of different volumes of HIIT on hypertensive patients, and there are few studies on resistance training, so, a more comprehensive analysis cannot be carried out; (6) There is almost no analysis of gender in the literature, so it is impossible to analyze the image of the effect of sports intervention from the perspective of gender; and (7) Only a few studies have conducted long-term follow-up on the effect of the intervention, and it is hard to analyze the long-term effect after the intervention.

## Conclusion

In the network meta-analysis, magnitudes of SBP and DBP (main indicators of blood pressure), BMI (index of obesity), rest HR, time to exhaustion, ventilatory thresholds, body fat, Max VO2, total cholesterol, max HR, and mean arterial pressure (index of another health status) of patients with hypertension are widely adopted to assess the health improvement. Taking the blood pressure level and the improvement of other health conditions after intervention as the main criteria, the effects of different volumes and intensity of physical exercise on the profile of people with hypertension were indirectly compared. The verified consistency model is applied to network meta-analysis. According to the results of our systematic review and web meta-analysis on patients with hypertension, MIT intervention is superior to other types of exercise in improving blood pressure, while HVHIIT is more effective in reducing BMI and rest HR. In addition, the effect of exercise on improving health status is different among different types of patients, suggesting that exercise with different volumes and intensity should be selected according to the severity of the disease. The use of antihypertensive drugs combined with the exercise intervention may lead to misjudgment. The results show that high-volume and appropriate-intensity exercise still has great potential in improving the health status of people with hypertension. However, there are few studies on the effects of different volume of HIIT and the other types of exercise on people with hypertension. More systematic well-planned studies are needed to evaluate the role of different volumes, intensity, and types of exercise training with long-term intervention in the improvement of the health of patients with hypertension.

## Author Contributions

ZL, YS, and YG: conceptualization. YG, HC, SL, and YS: methodology and validation. ZL and YG: writing-original draft preparation. YS, SL, and HC: writing-review and editing. E-CT and YG: supervision. All authors have read and agreed to the published version of the manuscript.

## Funding

This study was sponsored by the Major Program of the National Social Science Foundation of China (Grant number: 19ZDA352), the National Key R&D Programs of China (Grant number: 2018YFF0300903), Philosophy and Social Sciences Project of Zhejiang Province of China (Grant number: 22NDQN223YB), Public Welfare Science and Technology Project of Ningbo of China (Grant number: 2021S134), and K. C. Wong Magna Fund in Ningbo University.

## Conflict of Interest

The authors declare that the research was conducted in the absence of any commercial or financial relationships that could be construed as a potential conflict of interest.

## Publisher's Note

All claims expressed in this article are solely those of the authors and do not necessarily represent those of their affiliated organizations, or those of the publisher, the editors and the reviewers. Any product that may be evaluated in this article, or claim that may be made by its manufacturer, is not guaranteed or endorsed by the publisher.
